# Clinical evaluation of whole blood prothrombin time (PT) and international normalized ratio (INR) using a Laser Speckle Rheology sensor

**DOI:** 10.1038/s41598-017-08693-5

**Published:** 2017-08-23

**Authors:** Markandey M. Tripathi, Satoru Egawa, Alexandra G. Wirth, Diane M. Tshikudi, Elizabeth M. Van Cott, Seemantini K. Nadkarni

**Affiliations:** 1 Wellman Center for Photomedicine, Massachusetts General Hospital, Harvard Medical School, Boston, USA; 2 Department of Precision Engineering, University of Tokyo, Tokyo, Japan; 3 Department of Pathology, Massachusetts General Hospital, Harvard Medical School, Boston, USA

## Abstract

Prothrombin time (PT) and the associated international normalized ratio (INR) are routinely tested to assess the risk of bleeding or thrombosis and to monitor response to anticoagulant therapy in patients. To measure PT/INR, conventional coagulation testing (CCT) is performed, which is time-consuming and requires the separation of cellular components from whole blood. Here, we report on a portable and battery-operated optical sensor that can rapidly quantify PT/INR within seconds by measuring alterations in the viscoelastic properties of a drop of whole blood following activation of coagulation with thromboplastin. In this study, PT/INR values were measured in 60 patients using the optical sensor and compared with the corresponding CCT values. Our results report a close correlation and high concordance between PT/INR measured using the two approaches. These findings confirm the accuracy of our optical sensing approach for rapid PT/INR testing in whole blood and highlight the potential for use at the point-of-care or for patient self-testing.

## Introduction

Effective blood coagulation is a key physiological process to maintain hemostasis and prevent uncontrolled blood loss following injury. Impairments in coagulation, if inadequately treated, can cause excessive hemorrhage leading to organ failure and increasing the risk of mortality by five-fold in hospitalized patients^1–3^. On the other hand, excessive clotting can result in life-threatening thrombotic conditions such as deep vein thrombosis (DVT), pulmonary embolism (PE), myocardial infarction (MI) or stroke^4, 5^. Based on the coagulation defect, clinical management protocols include the transfusion of blood products to manage impaired coagulation and prevent dangerous blood loss^6, 7^, or the administration of anticoagulant agents to prevent thrombotic states^8–10^. To guide the appropriate transfusion strategy or permit effective anticoagulant dosing, an accurate assessment of the patient’s coagulation status is critical.

The laboratory assessment of prothrombin time (PT) and the associated international normalized ratio (INR) has been routinely used to determine a patient’s coagulation status and inform therapy. Traditionally, the PT is measured in platelet poor plasma (PPP) by activating coagulation via the extrinsic pathway using tissue factor (TF)^11^. From the measured PT value, the INR is further extracted as a standardized number that accounts for inter-device variations in PT measurements and the difference in the sensitivity of the TF activator^12^. However, given the requirement to transport and centrifuge the specimen, the CCT turnaround time is often too long (~1hr) to be reliable for informing treatment decisions particularly in the context of rapidly changing coagulation conditions in critically ill or injured patients. To address the need for rapid coagulation testing at the patient’s bedside, new approaches for measuring PT and INR in whole blood have been recently developed and commercialized^13^. Studies have shown that PT/INR testing at the point-of-care (PoC) or in the home setting significantly improves patient outcome by reducing the risk of hemorrhagic and thromboembolic complications^14, 15^. Lowering the cost of PT/INR testing in the hospital setting or for patient self-testing may likely further reduce overall health care costs associated with coagulation testing while potentially improving patient outcome^13, 16–19^.

We have previously reported on an optical approach that utilizes Laser Speckle Rheology (LSR) techniques to assess a patient’s coagulation status using a few drops of whole blood by measuring changes in blood viscoelastic properties during coagulation from a time series of laser speckle patterns^20, 21^. Laser speckle, a random intensity pattern that occurs by the interference of coherent light scattered from tissue, is exquisitely sensitive to the passive Brownian motion of intrinsic light scattering particles. During blood coagulation, the formation of a fibrin-platelet clot influences Brownian displacements of intrinsic light scattering centers, altering the rate of speckle intensity fluctuations. We have shown that the viscoelastic modulus of clotting blood can be quantified from the temporal intensity fluctuations of speckle patterns to derive information about blood coagulation status^20, 21^. Our previous work was limited to measuring activated thromboplastin times (aPTT) via the intrinsic pathway, and we observed an incidental (albeit lower) correlation with CCT values of PT. Moreover, clotting times spanned several minutes in our prior work in contrast to seconds measured by laboratory methods. Given the long measurement times, bulky LSR instrumentation, large blood volume (120 µL) and differences in diagnostic ranges compared with standard CCT values, the clinical utility of our prior study for PoC use was somewhat limited.

In the current study, we address the limitations of our prior work and demonstrate that LSR can assess a patient’s blood coagulation status with PT/INR values equivalent to laboratory values within a measurement time of a few seconds following the addition of thromboplastin reagent. Furthermore, we have designed and utilized a battery-operated, hand-held optical sensor for PT/INR testing that requires a just a drop of whole blood (40 μL) and an identical volume of thromboplastin reagent, that could potentially lead to rapid and inexpensive coagulation testing at the PoC or for patient self-testing^22^. Taken together, our prior work on measuring intrinsic coagulation and the current advances in this manuscript on measuring extrinsic coagulation in a drop of blood, provide sufficient information for accurately measuring both aPTT and PT times, necessary for moving the device forward towards clinical applicability.

## Results and Discussion

### PT/INR assessment using the LSR sensor

**Figure 1:**
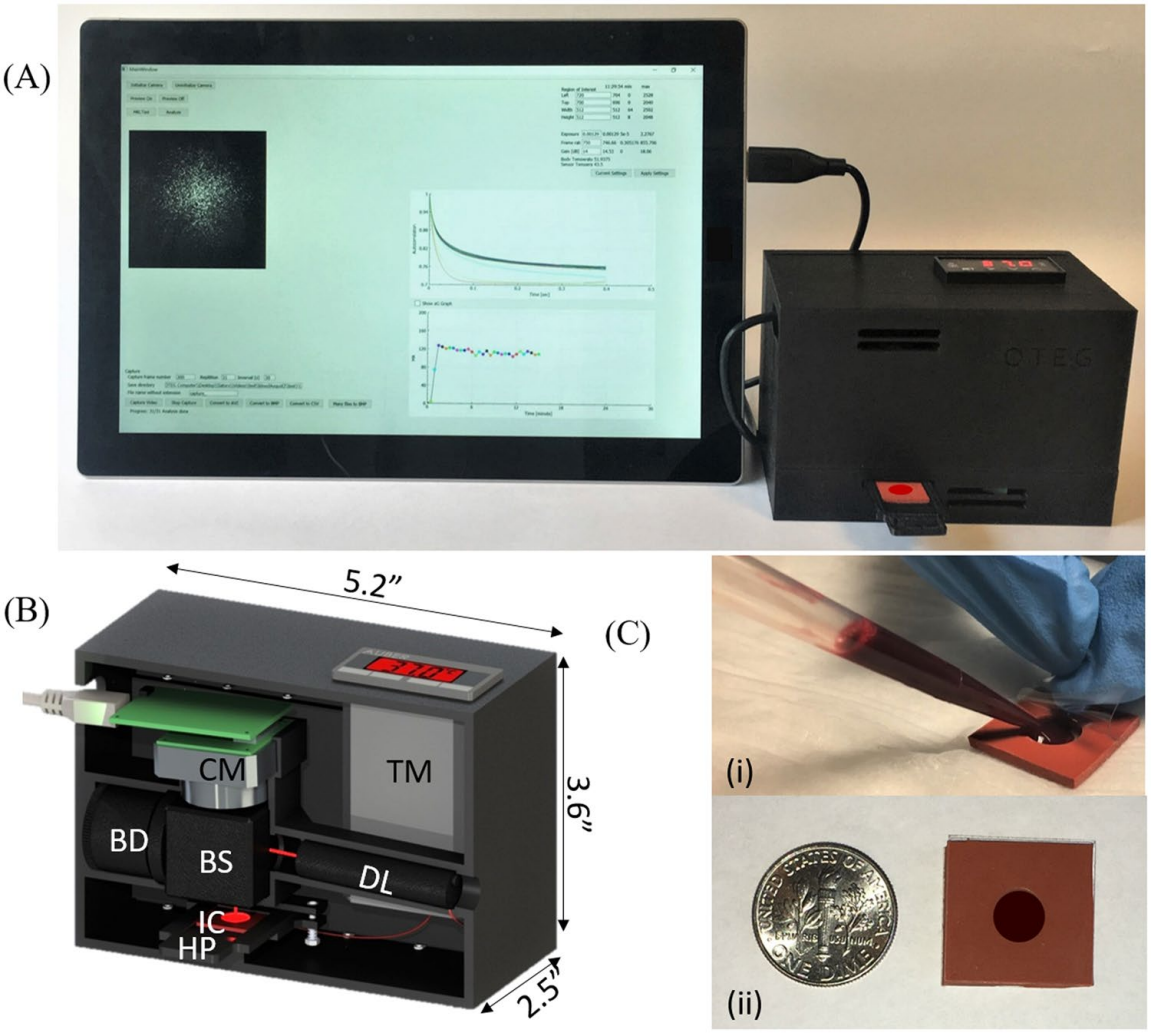
LSR-based coagulation sensor: **(A)** Photograph of the hand-held sensor and compatible Window surface tablet interface, **(B)** Computer aided diagram of the optical and mechanical device configuration. Light from a 690 nm diode laser (DL) was focused (spot size 100 μm) by a lens on the disposable test cartridge (IC) containing 40 μL of thromboplastin-activated whole blood. Cross-polarized laser speckle patterns were acquired at 180° back-scattering geometry via a beam-splitter (BS) using a USB CMOS camera (CM) equipped with an imaging optics consist of a linear polarizer, a 500 µm aperture and an f 9 mm focusing lens. Beam dump (BD) dumps the laser beam transmitted through the beam splitter (BS). A miniature heating element (HP), temperature controller (TM) and a custom cartridge tray was incorporated within the hand-held sensor. The captured speckle patterns were transferred to a Microsoft Surface^TM^ tablet computer for further processing. (**C**) Inexpensive test cartridges were fabricated by laser cutting a silicone base with a transparent polycarbonate film overlay to sample a drop of whole blood (40 μL).

**Figure 2:**
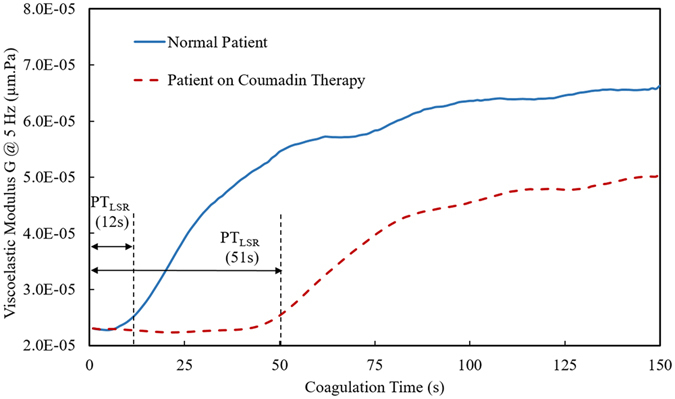
Viscoelastic modulus, G, at 5 Hz measured by the LSR sensor is plotted as a function of coagulation time from blood samples obtained from a normal patient (with reported normal laboratory PT/INR value) and a patient on Coumadin medication. The G trace measured in real-time during coagulation was analyzed to estimate the PT and INR values. The blood sample from normal patient showed shorter PT_LSR_ value (12 s) in comparison to the blood sample from the patient on Coumadin therapy (51 s). The corresponding PT_Lab_ values obtained from standard laboratory testing were 14 s and 43 s for the normal and Coumadin treated patient respectively.

As described in the Methods section (Fig. 1), we developed a hand-held LSR sensor to quantify patient’s PT/INR by analyzing laser speckle fluctuations emerging from a drop of blood sample mixed with thromboplastin reagent. The LSR sensor measured the viscoelastic modulus, G, of clotting blood at a frequency of 5 Hz from the temporal intensity fluctuations of speckle patterns captured during blood coagulation (See Methods section). Figure 2 shows the trend in G(t) measured by LSR following addition the thromboplastin reagent in two patient samples: a patient with PT_Lab_/INR_Lab_ within the normal range (blue solid line) and a patient on Coumadin therapy with elevated PT_Lab_/INR_Lab_ values (red dotted line). For the normal patient, a constant low value of G was observed during the early phase of coagulation (t < 12 s) followed by a rapid increase in G observed from 12 to 34 seconds owing to the conversion of soluble fibrinogen to fibrin and the initiation of a fibrin-platelet clot. In the plateau phase of G(t) curve (>34 s), no significant change in G was observed indicating completion of the clot formation process. The PT_LSR_ measured for the normal patient was 12 s, with a corresponding INR_LSR_ of 1.1 (PT_Lab_/INR_Lab_ = 14 s/1.1). On the other hand, for the blood sample obtained from the patient on Coumadin therapy, G remained at a constant low value for ~47 seconds after tissue factor activation and stabilized to a higher G value at ~90 seconds. The PT_LSR_ measured from the G curve for the Coumadin treated patient was 51 s with a corresponding INR_LSR_ of 4.6 (PT_Lab_/INR_Lab_ = 43 s/4.3). The delay in the rise of G for the patient on Coumadin therapy can be explained by the delay in the conversion of fibrinogen to fibrin, as it is well known that Coumadin inhibits the synthesis of the active form of vitamin K dependent pro-coagulation factors such as Factors II, VII, IX, and X^23^ which are essential for catalyzing fibrin formation. We further observed that the plateau modulus value of the G(t) curve was significantly lower for the Coumadin-treated patient compared to the normal patient which could be similarly be attributed to inhibited fibrin formation and polymerization which in turn likely lowers the clot stiffness reflected by the G value^24^.

In our prior work^21^, a high-speed camera (operating at ~480 frames/s) was used to capture the full extent of speckle fluctuations arising from the rapid rearrangement of light scatterers such as RBCs and platelets present in whole blood. Here, to address the need for low-cost PT/INR testing, we employed a CMOS camera that acquired speckle frames at low frame rates of 30 frames/s and conducted speckle acquisition at short exposure time (1.3ms). Using low exposure time limited speckle blurring within each frame and allowed the calculation of the speckle intensity auto-correlation curve, g_2_(t), at sub-sampled frequencies. To investigate the equivalence between the high-speed and low-frame rate speckle capture, we conducted experiments to evaluate the coagulation process at two frame rates of 480 and 30 frames/s. By maintaining the same exposure time (1.3 ms) in both cases, we observed that changes in G during clotting (frequency = 5 Hz) were nearly identical at high and low frame rates. It is clear therefore that the reduction in frame rate does not impact the capability to accurately quantify PT, provided the exposure time is maintained sufficiently short to prevent speckle blurring.

### Analytical performance of the LSR sensor

To assess the accuracy of PT/INR measurement, a quality assessment test was conducted. This was essential to investigate whether factors such as blood or thromboplastin reagent volume, temperature, activation time, and reagent quality could potentially influence PT/INR measurements. We tested three quality control specimens that are used for quality assessment of the conventional laboratory PT devices. The control specimens (TriniCHECK™, Tcoag, Wicklow, Ireland) were designated as Level 1 (normal, reference PT range 10–13 s), Level 2 (high, reference PT range 15–20 s), and Level 3 (high, reference PT range 22–28 s). LSR was conducted on all samples and experiments were conducted in triplicate. The results, provided in Table 1, show that for all the three control specimens, PT_LSR_ was within the three reference ranges, confirming the accuracy of LSR approach for measuring PT.

**Table 1:** Quality assessment test for LSR sensor.

	Reference PT Range	PT_LSR_
Level 1	10–13 Sec	(12.1 ± 0.6) Sec
Level 2	15–20 Sec	(17.3 ± 0.9) Sec
Level 3	22–28 Sec	(26.9 ± 1.3) Sec

The precision of the PT/INR measurement with LSR sensor was assessed. Two blood samples with reported normal (13.4 s/1.08) and high (27.2 s/2.52) PT/INR values were used for precision assessment (Table 2). Each blood sample was measured 10 times with the LSR sensor. To quantify precision, the coefficient of variation (CV) was calculated which was defined as the standard deviation divided by mean of the measured value. The estimated CV values for both normal and high PT/INR was less than 6% which was well within the expected range of commercially available PT analyzers for point-of-care use^25^.

**Table 2:** Repeatability testing of PT/INR measurement with LSR sensor.

	Normal PT/INR	High PT/INR
PT_LAB_	13.4	27.2
INR_LAB_	1.08	2.52
PT_LSR_, mean ± SD (%CV)	13.7 ± 0.6 (4.4%)	28.0 ± 1.4 (4.9%)
INR_LSR_, mean ± SD (%CV)	1.08 ± 0.05 (5.2%)	2.52 ± 0.14 (5.7%)

(n = 10 measurements per blood sample, %CV was calculated by dividing the SD by mean).

### Comparison of PT/INR values by LSR versus and standard laboratory testing

**Figure 3:**
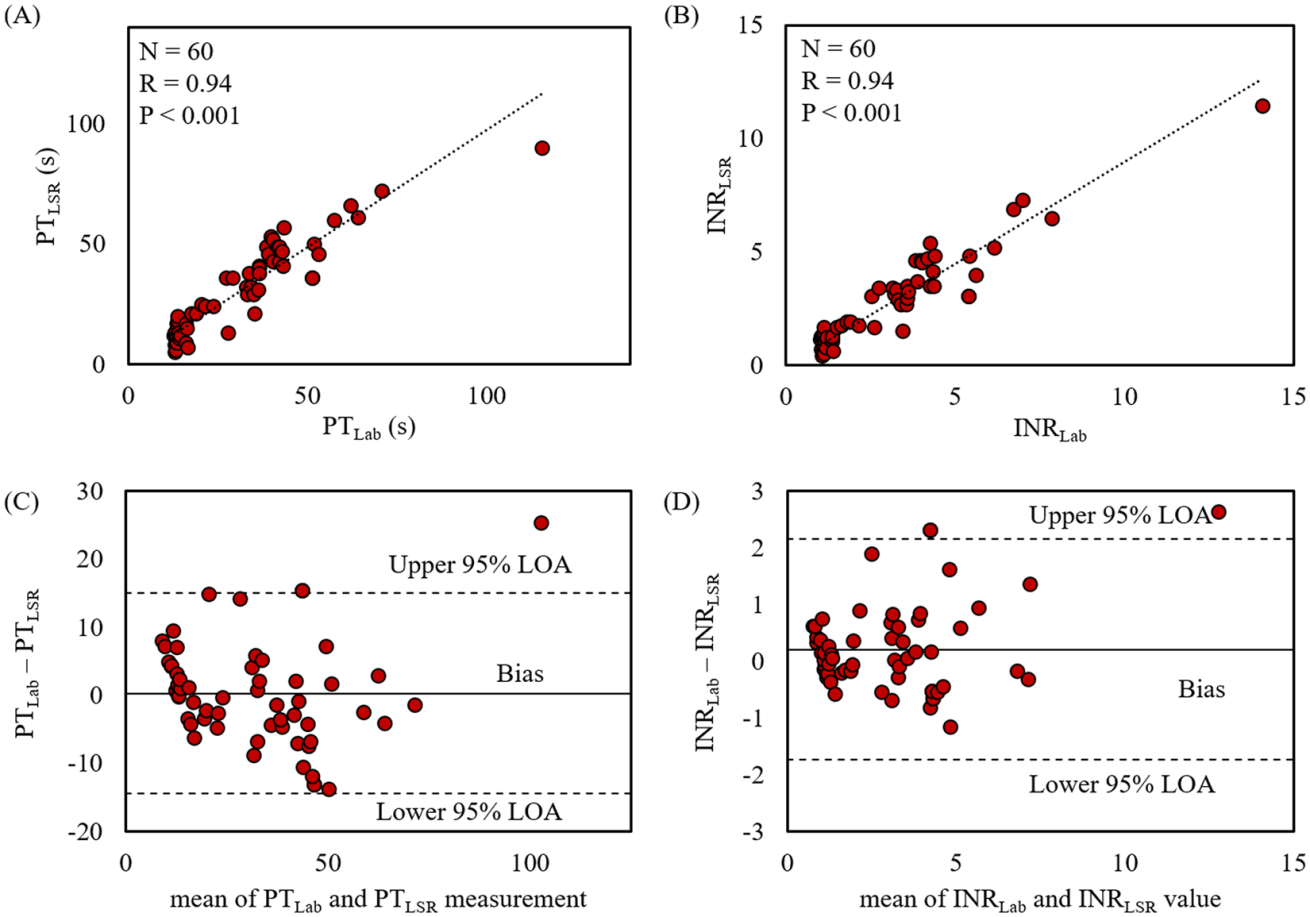
The plots show **(A)** PT and **(B)** INR measured using the LSR sensor against corresponding values obtained from standard laboratory tests measured from 60 patient whole blood samples. A strong statistically significant correlation between the two approaches (R = 0.94, p < 0.001) is observed. Bland-Altman plots comparing **(C)** PT and **(D)** INR measured with the LSR sensor and standard laboratory results show high concordance between both test measurements. Upper and lower dotted lines represent 95% limit of agreement (LOA) while the middle line represents bias that shows the average difference in the values measured with LSR sensor and laboratory test.

The 60 patient samples analyzed in this study had a large range of PT_Lab_/INR_Lab_ values, from 12.6 s/1.0 to 115.4 s/14.1, with a mean of 33.9 s/3.3 and a median of 35.1 s/3.4. For reference, the diagnostic normal range of the laboratory PT/INR is 11.0 s/0.9 to 14.0 s/1.1. In our study, 15 patients had PT_Lab_/INR_Lab_ within the normal range (11.0 s/0.9–14.0 s/1.1), 10 patients in the sub-therapeutic range (14.1 s/1.2–21.9 s/1.9), 5 patients in the therapeutic range (22.0 s/2.0–31 s/2.9) and 30 patients in the supra-therapeutic range (>31.1 s/ >3)^26^. In Fig. 3A and B, the PT_LSR_ and INR_LSR_ values reported using LSR from all 60 patients are plotted against the corresponding laboratory values. From the pooled data, it is evident that the PT_LSR_ and INR_LSR_ values exhibited an excellent correlation with laboratory results (R = 0.94, p < 0.001).

The equivalence between the LSR and laboratory-based approaches was further evaluated using a Bland Altman test by plotting the difference in the PT/INR values obtained by two methods against the mean value (Fig. 3C,D)^27^. High concordance was observed between PT_LSR_/INR_LSR_ and the corresponding CCT results, with majority of measurements falling within or at 95% limit of acceptance in Bland-Altman analysis (Fig. 3C,D). Based on the Bland-Altman analysis for PT measurement (Fig. 3C), the data set had a small bias of −0.3 s. The mean difference between PT_Lab_ and PT_LSR_ values was 2.6%, significantly below the clinically acceptable error (10%) for PT testing^28^. These small differences may be attributed to the differences in the thromboplastin concentration used in both methods to activate coagulation and the use of whole blood employed for LSR testing versus plasma in CCT^29^. Similarly, the INR value measured by LSR corresponded closely with CCT values, with only one out of 60 patient samples only slightly deviating above the upper 95% limit of agreement (LOA), and a small bias of 0.06. These observations suggest that the LSR sensor is nearly equivalent to the corresponding CCT approach in quantifying PT/INR, thus establishing the accuracy of our new approach. Several studies have demonstrated the enormous advantages of PT/INR self-testing that include improved patient satisfaction due to less-invasive fingerstick based blood collection (compared to venipuncture draws), rapid treatment adjustment due to immediate availability of results, improved anticoagulant drug adherence by patients and more frequent monitoring of coagulation status, which all contribute to more effective anticoagulation management that results in the reduced risk of hemorrhagic and thromboembolic complications^14, 15, 30^. However, current PT/INR testing at the PoC incurs a cost that is similar to or slightly higher than conventional laboratory testing, likely limiting in part, the widespread penetration of PoC PT/INR testing in hospitals or low resource settings^13, 19^. New approaches are being explored for PT/INR monitoring that include the use of quartz crystal^31^, magnetoelastic transducers^32^ and MEMS sensors^33^. The mechanical perturbation employed in these methodologies might however interfere with the clot formation process and therefore may not reflect the natural coagulation process *in situ*. Other optical methods have been proposed that present promising alternative strategies, however thus far these approaches utilize bulky bench-top instruments that may be unsuitable for PoC use^34–36^. In contrast to the above techniques, the LSR sensor described in this paper holds potential to provide rapid and cost-effective PT/INR testing for wide-spread use. Moreover, the non-contact coagulation sensing strategy applied in the LSR sensor (that does not interfere with the clot formation process) may provide more accurate PT/INR assessment that truly depict coagulation status in physiological condition.

Owing to its inherently high detection sensitivity to small changes in sample viscoelasticity, the LSR-based approach enables the accurate quantification of blood coagulation status in patients. Over 200 million PT/INR tests are conducted worldwide each year, placing a huge burden on health care resources. Low-cost devices such as LST for PT/INR testing at the point-of-care will likely play an important role towards managing hemostasis in patients while reducing the substantial costs associated with coagulation testing^37^. While we expect that LSR may likely improve patient outcome and lower health care costs for coagulation testing in the future, further clinical testing and cost analysis studies will be required before the true clinical and financial impact of low-cost PT/INR testing can be fully ascertained.

## Methods

### Blood specimen and coagulation assay preparation

The study protocol was approved by the Institutional Review Board of Massachusetts General Hospital (MGH). De-identified, excess discarded whole blood samples from 60 patients undergoing laboratory-based PT/INR testing per standard-of-care, were collected in a 0.105 M sodium citrate vacutainer system. Since the blood was drawn from patients for laboratory testing and only excess and discarded blood sample was collected (after proper de-identification), our study protocol did not require taking consent from the patients. All methods were performed in accordance with relevant guidelines and regulations. To measure PT/INR using LSR, lyophilized thromboplastin from cultured human cells (TriniCLOT^TM^ PT HTF, Stago, Asnieres, France) was used as the blood coagulation activator, in the same manner as is used for standard laboratory testing of PT/INR. Thromboplastin derived from different sources may have different sensitivity in activating coagulation, which is often the source of variability between different laboratory instruments that measure absolute values of PT times. Therefore, thromboplastin used in this study for both LSR and CCTs was from the same batch so that the PT results could be directly compared. However, by calculating the corresponding INR values (detailed below), batch variations in the thromboplastin reagent can be accounted for in future clinical studies. For experiments, the thromboplastin formulation was reconstituted by adding 20 mL of deionized water and gently inverting 10 times to ensure complete rehydration. The reconstituted thromboplastin reagent was stored for at least 30 minutes to ensure stabilization and inverted gently before each test to ensure homogeneity. After one time preparation, the reconstituted PT reagent was stored at 2 to 8 °C for a duration of 10 days at a time (based on the specified stability period as per manufacturer’s specifications). To conduct LSR testing, 40 µL of citrated whole blood was loaded into a disposable, custom-fabricated test cartridge (described below) followed by the addition of 40 µL of the thromboplastin reagent (that contained calcium ions for re-calcification). The test cartridge was immediately loaded in the portable LSR device for measurement. The standard laboratory PT/INR testing also used TriniCLOT^TM^ PT HTF as the reagent, on a Destiny Max coagulation analyzer (Stago, Asnieres, France). The standard laboratory required 2.8 mL of whole blood for each PT/INR test compared to 40 µL for LSR. All PT experiments were conducted within 4 h following blood collection to reduce experimental variations^38^.

### Whole blood coagulation sensing using the LSR sensor

The portable LSR sensor used for PT/INR measurement is shown in Fig. 1(A–C). Linearly polarized laser light (690 nm, 10 mW) from a diode laser (Newport Corp., LPM690–30C) was focused to a 100 µm spot on the surface of the test cartridge containing the blood sample. Backscattered and cross-polarized laser speckle patterns, reflected from the sample through a beam splitter and a 90^0^ rotated linear polarizer, were collected by a miniature board-level CMOS camera (PixelLink, PL-D725MU-BL, 5.3MP Mono, Board Level, USB 3.0) equipped with an imaging lens system. The imaging optics, which consisted of a linear polarizer, a 500 µm aperture and a plano-convex lens, were mounted and secured in place using custom 3D printed mounts. The magnification and F-number of the lens system were designed to ensure the speckle-to-pixel ratio of at least 2 for an adequate spatial sampling of each speckle spot. All components of the sensor were contained within a custom-designed, 3D-printed chassis. A cartridge tray was designed and printed to load and secure the test cartridge loaded with blood during the measurement. The blood sample was maintained at 37 °C using a low-profile temperature controller (Aubur Instruments, SYL-1612B), surface heating element (Omega, KHLV-101/5-P) and a miniature resistance thermometer (Aubur Instruments, Pt100MN) incorporated within the cartridge tray. The LSR sensor (dimensions: 5.2″ × 3.6″ × 2.5″) weighed less than 1 lb and could easily be hand-held to permit portability. In the current instrument, 6 × 3.7 V batteries were used with a 3400 mAh current rating to ensure all day (8 hours) operation. Our future prototypes will be developed with energy efficient laser sources, temperature controllers and low-frame rate cameras to reduce the power requirement to less than 10^th^ of the current power needs.

During measurement, speckle patterns collected from the blood sample were acquired at 30 frame/s from the imaging region of interest (ROI) covering 8 × 8 mm area on 512 × 512 pixels of the CMOS sensor (5.5 × 5.5 μm pixel size) for a maximum duration of 150 seconds during the coagulation process. The CMOS camera was connected to a portable Microsoft Surface^TM^ tablet computer via a USB 3 port to accomplish real-time image transfer at the rate of 20 MHz and perform speckle image analysis to compute and report PT/INR values in near real-time. The disposable test cartridges (Fig. 1C) were custom-fabricated in-house by laser cutting a small chamber (radius = 3mm) within a blood-compatible silicon base (L = 2 cm, W = 2 cm, D = 0.28 cm). The cartridge is enclosed on both sides with an optically clear polycarbonate sheet (thickness = 0.15mm) to hold the blood sample and provide an optically clear window to conduct PT/INR measurement. Blood and the activator is placed in the cartridge by removing the clear top sheet and after loading, the top sheet is placed back to seal the cartridge (Fig. 1C(i)).

### PT/INR analysis

The LSR sensor evaluated prothrombin time from the temporal evolution of the viscoelastic modulus of clotting blood measured at 1 second intervals from the time-series of laser speckle patterns using algorithms that were previously well described^20, 21, 39, 40^. Briefly, to calculate the viscoelastic modulus of blood, G, the speckle intensity autocorrelation curve, g_2_(t), was first calculated by performing a 2-dimensional cross-correlation analysis between first speckle frames with subsequent frame of the speckle image time series as^20, 21, 39–41^:

1 $${{\rm{g}}}_{2}({\rm{t}})={\langle \frac{{\langle {{\rm{I}}({\rm{t}}}_{0}{){\rm{I}}({\rm{t}}}_{0}+{\rm{t}})\rangle }_{{\rm{pixels}}}}{\sqrt{{\langle {{\rm{I}}({\rm{t}}}_{0}{)}^{2}\rangle }_{{\rm{pixels}}}{\langle {{\rm{I}}({\rm{t}}}_{0}+{{\rm{t}})}^{2}\rangle }_{{\rm{pixels}}}}}\rangle }_{{{\rm{t}}}_{0}}$$

Here I(t_0_) and I(t_0_ + t) defines the speckle intensities at times t_0_ and t_0_ + t, and <  > pixels and <  > t_0_ indicates spatial and temporal averaging over all the pixels (512 × 512) and for the duration of speckle time series (1 s) respectively. The measured autocorrelation values were used to estimate the mean square displacement (MSD) of light scattering particles by exploiting the conventional diffuse wave spectroscopy (DWS) formulism based on diffusion approximation^20, 39–43^. As previously described^20, 39–41^, the MSD quantifies the random Brownian diffusion of scattering particles in a viscous medium and is related to its viscoelastic modulus, G*(ω), through the Generalized Stokes-Einstein Relation (GSER) as follows^42–46^:

3 $${{\rm{G}}}^{\ast }(\omega )=\frac{{{\rm{K}}}_{{\rm{b}}}{\rm{T}}}{a\pi \langle {{\rm{\Delta }}r}^{2}(1/\omega )\rangle {\rm{\Gamma }}[1+\alpha (\omega )]}$$

where ${\text{K}}_{\text{b}}$ is the Boltzmann constant (=1.38 × 10^−23^), and T is the temperature in Kelvin (=310 ° K), ω (=2πυ = 1/t) represents the angular frequency, υ represents frequency, t is time in sec, Γ denotes the gamma function and ${\alpha }{(}{\omega }{)}$ = ${|\frac{{dln}\langle {\rm{\Delta }}{{r}}^{{2}}(t)\rangle }{d\mathrm{ln}t}|}_{t=1/\omega }$ denote MSD slope in a log-log plot^47^. To compute the absolute value of the viscoelastic modulus, G*(ω), via the GSER, knowledge of the particle radius, a, of light scattering particles is required. During coagulation, however, the effective radius of light scatterers is consistently altered with the formation of fibrin monomers and due to platelet aggregation. As a result, an accurate estimate of ‘a’ is difficult to obtain. Instead, we measured the quantity G at a frequency of ω = 5 Hz to indicate clot viscoelasticity, where G = a × $|{{\rm{G}}}^{\ast }({\rm{\omega }})|$, was equal to the product of the viscoelastic modulus and the particle radius, a. Using this approach, we have previously established that LSR can accurately quantify the time course evolution of the viscoelastic modulus during the process of blood coagulation^20^. Next, to calculate the prothrombin time, the time course of the modulus, G, was first displayed as a function of coagulation time, t (Fig. 2). We then calculate the first derivative of the G(t) curve during whole blood coagulation and the time at which the derivative drops to 2% of the maximum value was recorded as the PT_LSR_ value (Fig. 2). The corresponding INR_LSR_ value was then calculated using published methods^11^. Each PT_LSR_ value was first divided by the average of all PT_LSR_ values of patients within the normal laboratory diagnostic range (PT_Lab_ = 11–14 s) and then the ratio was raised to a power designated as international sensitivity index (ISI). In our study, the mean PT value for normal patients measured with LSR sensor was 16.0 s and the ISI value of the thromboplastin used in the study was 1.19 (as reported by the manufacturer). All of the above steps of the algorithm were completed within seconds using a Microsoft Surface tablet, and the PT_LSR_/INR_LSR_ value for each patient was reported in less 30 seconds following completion of the test. For all patients, PT_LSR_/INR_LSR_ values calculated using the portable LSR sensor were compared with the corresponding laboratory PT_Lab_/INR_Lab_ results obtained as clinical standard-of-care.

### Quality assessment experiments

Lyophilized human plasma samples with similar characteristics as human plasma, TriniCHECK™ Level 1, Level 2, and Level 3 (Tcoag, Wicklow, Ireland) were used for quality assessment. Control specimens were reconstituted with 1.0 ml of purified water, swirled gently and kept for 20 minutes at room temperature (18–25 °C) to assure complete hydration. Next 60 µL of reconstituted plasma was mixed with 20 µL of thromboplastin reagent in the test cartridge and immediately measured with the LSR sensor. During analysis, PT time was similarly calculated from the first derivative of the G(t) curve as described above.

### Statistical analysis

The LSR values of PT_LSR_/INR_LSR_ were compared with the corresponding laboratory values using linear regression analysis. The agreement between the absolute values of PT/INR measured by both approach was tested using the Bland Altman analysis (using Prism software, GraphPad, San Diego, CA). In all cases, P < 0.05 was considered statistically significant.
